# BCR Signaling Inhibitors: an Overview of Toxicities Associated with Ibrutinib and Idelalisib in Patients with Chronic Lymphocytic Leukemia

**DOI:** 10.4084/MJHID.2016.011

**Published:** 2016-02-10

**Authors:** Lorenzo Falchi, Jessica M. Baron, Carrie Anne Orlikowski, Alessandra Ferrajoli

**Affiliations:** 1 Department of Medicine, Division of Hematology/Oncology, Columbia University Medical Center, New York, NY; 2 Department of Leukemia, The University of Texas MD Anderson Cancer Center, Houston, TX

## Abstract

The B-cell receptor (BCR) signaling inhibitors ibrutinib and idelalisib are revolutionizing the treatment of chronic lymphocytic leukemia (CLL) and other B-cell malignancies. These oral agents, both alone and in combination with other drugs, have shown remarkable clinical activity in relapsed or refractory CLL across all risk groups, and have been approved by the Food and Drug Administration for this indication. Preliminary data suggest that an even greater benefit can be expected in treatment-naïve CLL patients. Both ibrutinib and idelalisib are well tolerated by most patients, including older, frailer individuals. Toxicities are usually mild and self-resolving. Clinicians must, however, be aware of a number of peculiar adverse events, the effects of which can be severe enough to limit the clinical use of these agents. In this review, we survey the salient aspects of the pharmacology and clinical experience with the use of BCR signaling inhibitors for the treatment of patients with CLL. We next focus on both the most common and the most clinically significant toxicities associated with these drugs.

## Introduction

Chronic lymphocytic leukemia (CLL) is the most common leukemia occurring among adults in the Western world.^1^ It is typically diagnosed in advanced age, and is frequently discovered accidentally in otherwise asymptomatic individuals. Its clinical course tends to be indolent; 5-year survival exceeds 80% according to the latest Surveillance, Epidemiology, and End Results (SEER) Report.^2^ While early-stage asymptomatic CLL can be managed expectantly, most patients require therapy during the course of their disease and can achieve long periods of complete clinical remission (CR) with current treatment options.^3^

CLL therapy has historically been based on the use of alkylating agents, such as chlorambucil, which did not have an impact on the natural history of the disease.^4^ With the advent of fludarabine-based chemotherapy, patients experienced longer disease-free survival, but did not see an improvement in overall survival (OS).^5^ The addition of rituximab (R) to fludarabine-cyclophosphamide (FC) chemotherapy (FCR) improved survival relative to FC alone in fit patients with relapsed or refractory,^6^ as well as treatment-naïve CLL.^7^ Therefore, FCR represents the standard of care for fit patients with newly diagnosed CLL in need of treatment.

Recent advances in the understanding of the pathobiology of CLL have paved the way for the discovery and clinical development of novel targeted agents for its treatment. In particular, drugs that interfere with the B-cell receptor (BCR) signaling pathway have represented a major breakthrough, and are rapidly changing the therapeutic landscape in CLL. These agents have shown impressive clinical activity in both heavily pretreated and treatment-naïve patients, including those with high-risk features namely bulky disease, fludarabine refractoriness, 17p deletion [del(17p)].^8^

Clinical experience has hitherto shown that these new agents are well tolerated across all patient subsets, including elderly and/or unfit individuals. The most common side effects are generally mild (grade 1–2), and rarely lead to treatment discontinuation or serious adverse events (AE).

In this review, we provide a summary of the pharmacology and clinical activity of two BCR signaling inhibitors, ibrutinib and idelalisib; we follow with a discussion of the characteristics and management of the most common treatment-emergent AE so far reported in patients with CLL. Ibrutinib and idelalisib are included in the most recent guidelines for the treatment of CLL and have been approved by the Food and Drug Administration (FDA) and the European Medicine Agency (EMA).

## Mechanism of Action, Pharmacokinetics, and Pharmacodynamics

### Ibrutinib

Ibrutinib is a Tec kinase inhibitor that irreversibly blocks Bruton’s tyrosine kinase (BTK) by covalently binding cysteine-481 in the BTK active site.^9^,^10^ BTK is found downstream of the BCR and plays a crucial role in B-cell development and signaling. The early placement of BTK in this cascade causes it to be an integral part of many functions of the BCR. Activation of BTK elicits continuation of the cell cycle via nuclear receptor of activated T cells (NFAT), nuclear factor-κB (NFκB), and extracellular signal-regulated kinases (ERK) pathways, leading to increased transcriptional activity, proliferation, and survival.^11^,^12^ In CLL cells, BTK is uniformly overexpressed and causes constitutive activation of these pathways.^13^ Furthermore, BTK is involved in B-cell adhesion, chemotaxis, and migration to lymph nodes via CXCL12 and CXCL13 activation of the chemokine receptors CXCR4 and CXCR5.^10^,^14^,^15^ By inhibiting BTK, ibrutinib inhibits CLL cell migration, thus preventing exposure to further activation, survival and proliferation signals from the lymph node microenvironment.^12^ Conversely, it has been suggested that ibrutinib could cause CLL cells already within lymph node proliferation centers to be expelled into circulation.^16^

Ibrutinib is rapidly absorbed after oral dosing, reaching peak levels in 1–2 hours. A dose-dependent increase in exposure is seen with up to 840 mg daily.^10^ Although ibrutinib may be administered without regard to food, it is important to note that its pharmacokinetics (PK) can vary if food is ingested between 30 minutes prior to 2 hours following the dose, particularly if the food is high in fat. These changes are thought to be due to the increase in intestinal blood flow. With food, peak serum concentration (C_max_) is increased 2- to 4-fold, and area under the curve (AUC) is increased approximately 2-fold; consequently, exposure during fasting state is about 60% of that seen with food. Time to peak levels (T_max_) is also increased, to 4 hours, under fed conditions.^17^ At a dose ≥2.5mg/kg/day, BTK was occupied for up to 24 hours at >90%.^10^ Ibrutinib is highly protein-bound (97.3%) with no concentration dependence between 50–1000 ng/ml and an apparent volume of distribution at steady state of about 10,000 liters.^10^ One study has shown that ibrutinib can cross the blood-brain barrier (1–7% of the dose found in cerebral spinal fluid).^18^ Ibrutinib is primarily eliminated through metabolism via CYP3A4, and, to a lesser extent, CYP2D6. Its metabolite, PCI-45227, also inhibits BTK, but with 15 times lower activity. Both ibrutinib and PCI-45227 are weak inducers of CYP450 isoenzymes. Approximately 80% of the dose is excreted through the feces. Due to high first-pass effect, clearance is about 2000 liters/h when fasting and 1000 liters/h when taken with food.^10^ Half-life elimination is approximately 4–6 hours.^10^,^17^

Neither age nor gender appear to affect ibrutinib PK. Population studies also suggest that weight and race (white versus black) may not significantly affect clearance.^10^,^17^,^19^ Strong CYP3A4 inhibitors increase the AUC of the drug by more than 10-fold; strong CYP3A4 inducers decrease the AUC by an equivalent amount. Both should be avoided, or ibrutinib should be held, if a strong CYP3A4 inhibitor must be given. If moderate CYP3A4 inducers or inhibitors must be given, it is recommended that the dose of ibrutinib be decreased to 140 mg daily until the inhibitor or inducer is discontinued.^10^

### Idelalisib

Idelalisib inhibits phosphatidylinositol 3-kinase (PI3K), a cytoplasmic tyrosine kinase involved in a number of signaling pathways within B-cells, including those downstream from the BCR and CD40. PI3K is involved in phosphorylation of phosphatidylinositol 3,4,5-trisphosphate (PIP3), which in turn takes part in AKT and BTK activation. These pathways affect cell proliferation, survival, and migration.^20^–^22^ Specifically, idelalisib inhibits the δ isoform of PI3K found in hematopoietic cells, an isoform believed to be overactive in B-cell malignancies. In addition to blocking BCR signaling, inhibition of PI3Kδ is believed to interrupt CXCR4 and CXCR5 signaling and subsequent CLL cell homing, causing redistribution of these cells into circulation, removal from the lymph node microenvironment’s pro-survival signals and sensitization to apoptosis.^20^,^23^,^24^

Idelalisib is also dosed without regard to food. In fasting state, median T_max_ is 1.5 hours. When given with high-fat meals, the AUC is increased by 1.4-fold. Idelalsib’s exposure is not proportional to dose changes between 50 to 350 mg in fasting patients.^20^ Due to the consistent responses, lack of significant increased exposure with doses over 100 mg, and better exposure control with twice daily dosing, 150 mg twice daily was chosen as the recommended dose for clinical use.^25^ Idelalisib is highly protein-bound (84%) without concentration dependence, and has a volume of distribution at steady state of 23 liters. Its metabolism to inactive metabolites occurs in the liver primarily via aldehyde oxidase and CYP3A4, and to a lesser degree by UGT1A4. The half-life elimination for idelalisib is 8.2 hours, with a systemic clearance rate of 14.9 ml/hr. Idelalisib is mostly (78%) eliminated with the feces.^20^

According to population analyses, none of age, race, gender, or weight appear to affect the PK of idelalisib. Dose adjustment is recommended in hepatic dysfunction, as PK studies have shown AUC increases of up to 1.7-fold in patients with abnormal hepatic laboratory parameters. While adjustment does not seem to be required in renal impairment, idelalisib PK were not studied in patients with a creatinine clearance of <15 ml/min.^20^

*In vitro,* idelalisib is a strong inhibitor of CYP3A4, and also inhibits each of the following: CYP2C8, CYP2C19, UGT1A1, P-glycoprotein, OATP1B1 and OATP1B3. Co-administration of CYP3A4 substrates should be avoided in patients receiving this agent. Idelalisib also induces CYP2B6 and CYP3A4. Strong CYP3A4 and p-gp inducers can decrease idelalisib AUC by about 75%. Although strong inhibitors of CYP3A4 and p-gp do not seem to have as profound an effect on plasma levels, some studies have shown increased idelalisib AUC by 1.8-fold when administered after ketoconazole.^26^ Therefore, although patients should be monitored for idelalisib toxicities, the concomitant use of such inhibitors does not require dose adjustment.^20^

## Summary of Clinical Experience

### Ibrutinib

Ibrutinib is FDA-approved for the treatment of patients with relapsed or refractory CLL, or as frontline therapy in patients with del(17p) CLL. In a phase Ib/II study, 85 patients with relapsed or refractory CLL were treated with ibrutinib 420 mg daily (51) or 840 mg daily (34). Results for the two patient groups were similar, leading to premature closure of the second cohort and adoption of the 420 mg daily dose for subsequent studies. The overall response rate (ORR) was 91% and was independent of clinical or biological risk factors. The estimated 26-month progression-free survival (PFS) and OS were 75% and 83%, respectively.^27^ In the same trial, 29 previously untreated patients ≥65 years were also enrolled. After a median follow-up of 22 months, ORR was 71% and complete response rate (CRR) 13%. Only one patient progressed after 9.6 months and subsequently died at the time of the original publication.^28^ A recent 3-year follow-up analysis showed improved quality of response and sustained remissions (84% ORR and 23% CRR, for previously untreated patients; 90% ORR and 7% CRR, for patients with recurrent CLL). Progression occurred mainly in patients with relapsed del(17p) and/or del(11q) CLL.^29^ In a randomized, open-label, multicenter, phase III study, ibrutinib was compared with ofatumumab in patients with recurrent CLL. The median follow-up at the time of data analysis was 9.4 months. Ibrutinib was superior to ofatumumab both in terms of PFS, the primary endpoint (not reached vs. 8.1 months), and OS (90% vs. 81% at 12 months). Clinical benefit was also observed in patients with del(17p).^30^

A phase II study was conducted in 51 patients with CLL and del(17p) or *TP53* mutations (16 previously treated and 35 treatment-naïve). All but one treatment-naïve patient achieved an objective response. After 24 months, 9% of treatment-naïve and 20% of previously treated patients progressed; the estimated OS rates were 84% and 74%, respectively.^31^ In a separate phase II study of 144 patients with del(17p) CLL, the ORR was 83% after a median follow-up of 11.5 months and 12-month PFS and OS rates were 79% and 84%, respectively.^32^ Overall, high-risk patients appear to have inferior survival compared to those with a lower-risk disease, but their outcome after ibrutinib seems superior to that achieved with most other currently available therapies.

Ibrutinib was also tested in combination with other agents, including monoclonal antibodies and conventional chemotherapy. In a phase II study, 40 patients with high-risk CLL (del(17p), del(11q) or PFS <36 months after chemoimmunotherapy) were treated with a combination of ibrutinib 420 mg daily and rituximab (first weekly for 4 infusions, then monthly up to 6 cycles), followed by continuous ibrutinib until disease progression or unacceptable toxicity. ORR was 87%, including 8% CRR. The 18-month PFS was 78% (72% in patients with del(17p) or *TP53* mutation).^33^ Similarly, the combination of ibrutinib and ofatumumab (12 doses) was evaluated in 71 heavily pretreated patients with CLL/Small Lymphocytic Leukemia, 44% of whom had del(17p). ORR was 71–100% and estimated 12-month PFS 75–89%.^34^ In a multicenter phase Ib study, 30 patients with relapsed/refractory CLL received bendamustine and rituximab (BR) for up to 6 cycles combined with ibrutinib administered continuously until progression or unacceptable toxicity. Best ORR was 97%, including 40% best CRR, for a 36-month PFS of 70%.^35^ More recently, the results of a phase III randomized trial of BR with or without ibrutinib in previously treated CLL was reported. In that study, 578 patients with previously treated CLL were randomized to receive BR for 6 cycles or BR plus ibrutinib. ORR and CRR favored the BR plus ibrutinib arm. At a median follow-up of 17 months, the PFS (primary endpoint) was 13.3 months for BR, and was not reached for BR plus ibrutinib. The risk of progression or death was reduced by 80% in the latter group.^36^

Although ibrutinib is not currently approved for the treatment of all patients with newly diagnosed CLL, the results of a recently published randomized phase III study of ibrutinib versus chlorambucil in patients 65 years or older, should be mentioned. In this trial (N = 269), patients treated with ibrutinib had a higher response rate compared with those receiving chlorambucil (86% vs. 35%) and, after a median follow-up of 18.4 months, longer PFS (not reached vs. 18.9 months) and a reduction in the risk of death of 84%.^37^

### Idelalisib

Idelalisib is approved in combination with rituximab for the treatment of patients with relapsed or refractory CLL. In a phase I study including 54 patients treated with continuous idelalisib at varying doses and schedules, the ORR was 74% (all partial responses). At a dose ≥150 mg twice daily, the median PFS was 32 months (as compared with 7 months for patients receiving a lower dose). Median OS was not reached at the time of data analysis (36-month OS was 75%). Fifty-eight percent of the 13 patients with del(17p) responded to therapy, with a median PFS of 3 months.^38^

A subsequent phase III randomized trial of idelalisib and rituximab vs. placebo and rituximab (8 doses in both groups) in patients with relapsed CLL showed response rates of 81% vs. 13%, respectively. Again, no patient achieved CR. Combined idelalisib and rituximab achieved significantly higher 24-week PFS (93% vs. 46%) and 1-year OS (92% vs. 80%).^39^

## Ibrutinib: Safety Considerations

Ibrutinib discontinuation rates in the pivotal phase I study were 4% in the 420-mg dose group and 12% in the 840-mg one.^27^ A pooled analysis of the same study along with a phase I trial of ibrutinib in treatment-naïve elderly patients revealed that 81% of newly diagnosed patients and 53% of those with relapsed or refractory disease remained on study at a 3-year follow-up. AE were the primary reason for discontinuing therapy in 17 patients (13%), with only 4 cases having AE possibly related to ibrutinib treatment. Dose reductions due to AE were required in 13 patients. Both dose reduction and treatment discontinuation occurred primarily during the first year of treatment.^29^ In the phase III trial, dose reduction due to AE occurred in 4% of patients treated with ibrutinib, with diarrhea being the most common reason. Discontinuation of treatment because of AE was reported in an additional 4% of cases.^30^

Rates of discontinuation did not appear to increase significantly when ibrutinib was combined with other drugs. Indeed, 11% in patients treated with ibrutinib and ofatumumab discontinued,^34^ as did 7% of those receiving ibrutinib and BR. In the latter study, the full 6-cycle course of the BR component was not administered in 6 patients (20%).^35^

A recent pooled analysis of 308 patients enrolled in prospective clinical trials aimed to determine the reasons for discontinuation of ibrutinib. After a median follow-up of 20 months, 232 patients (75%) remained on therapy. Approximately 60% of those who discontinued the drug did so for reasons other than disease progression (28 due to infection, 8 due to other AE, 9 for other reasons), mostly during the first year of therapy. Age was the only independent risk factor for non-relapse discontinuation.^40^ An overview of common and relevant AE in patients treated with ibrutinib, alone or in combination with other agents, is offered in Table 1, and discussed below.

### Common AE

Diarrhea is one of the most common ibrutinib-related AE. It is thought of as an off-target effect, resulting from the blockade of other tyrosine kinases, such as the epidermal growth factor receptor.^41^ Diarrhea of any grade was recorded in the majority of patients across studies; it generally occurred within the first 4 weeks of therapy, was mild in severity (grade ≤2, that is 6 or less bowel movements a day), and self-limiting within about 3 weeks (without therapy). Severe diarrhea did not cause dose reductions, hospitalization, or treatment suspension in the majority of patients.^27^–^31^,^33^–^35^,^37^ In the follow-up analysis of the pivotal phase I study, around 60% of patients had at least 1 episode of diarrhea. Few patients had grade 3 diarrhea (≥7 bowel movements a day) and were successfully treated with antimotility agents, 2 patients had dose reductions, and 1 discontinued therapy.^29^ Low rates of severe diarrhea were observed in the phase III trial (4%).^30^

Fatigue is another frequently encountered AE during ibrutinib therapy. It is generally both mild and self-resolving without the need to hold therapy. Fatigue never constituted a reason for treatment discontinuation in clinical trials.^27^,^28^,^30^,^31^,^33^–^35^,^37^ Moreover, the frequency of fatigue was shown to decrease over time.^29^ This decreasemight be related, at least partially, to a progressive decrease in tumor burden and, consequently, circulating cytokine levels. Interestingly, Burger and colleagues reported that patients receiving ibrutinib combined with rituximab had an overall improvement of their quality of life (including fatigue) after 6 and 12 months of therapy.^33^

Although ibrutinib has been shown to ameliorate the symptoms of autoimmune arthritis,^41^ arthralgia (with or without joint swelling) is a common occurrence in patients treated with this agent; it can be associated with significant discomfort, and, in some cases, result in treatment discontinuation.

Tyrosine kinase receptors are known key regulators of lens clarity and organization.^42^,^43^ A concern was that corneal opacification or cataract formation might occur on ibrutinib via a tyrosine kinase mediated-mechanism. In the phase III study of ibrutinib vs. ofatumumab, the incidence of blurred vision was 10% vs. 3%, and of cataracts was 3% vs. 1%, respectively.^30^ Among 506 patients with B-cell cancers treated with single-agent ibrutinib, cataracts were reported in 2.6% of cases, which is consistent with the background rate in an age-matched population.^44^

### AE of Special Interest

#### Bleeding

Hemorrhage can be a serious, life-threatening AE. Ibrutinib may cause platelet dysfunction as an on-target effect. BTK is present on platelets and is required for collagen- or shear stress-induced platelet aggregation.^45^,^46^ Long-term BTK inhibition is also associated with increased megakaryocytes and giant platelets in peripheral blood, potentially leading to additional platelet dysfunction. *Ex vivo* experiments showed that ibrutinib at clinically achievable concentrations inhibited platelet signaling downstream of the collagen receptor glycoprotein VI and interfered with platelet adhesion on von Willebrand factor under arterial flow.^47^,^48^ These effects were corrected with the addition of platelets not exposed to ibrutinib or after treatment cessation (due to physiological platelet turnover).^48^ Another study confirmed these data on light transmission aggregometry and found a correlation between the degree of BTK inhibition and the occurrence of clinical bleeding (P = 0.044).^49^

An increased rate of serious hemorrhage, mainly intracranial and gastrointestinal bleeding, has been observed in patients with CLL treated with ibrutinib. In the long-term follow-up analysis of the phase I studies, a total of 61% of patients had experienced bleeding of any grade, with 10 patients having major hemorrhage. One patient died, albeit in the setting of multi-organ failure. Half of the untreated and about a third of previously treated patients were on aspirin during the study; 10% and 26%, respectively, were also on anticoagulants.^29^ Similarly, in the study from Jaglowski and colleagues, 7 patients experienced major hemorrhage, one of which was fatal. Three of these patients (including the one who died) were taking warfarin at the time of the AE.^34^ As safety data became available, prophylactic measures were incorporated into clinical trials, in order to decrease the risk of this complication. In the phase III study, patients who were on warfarin (but not other anticoagulants) were excluded, and a recommendation was made that ibrutinib be held for at least 3 to 7 days pre- and post-surgery, depending upon the type of surgery and the risk of bleeding. As a result, although bleeding AE of any grade were more common in the ibrutinib group than in the ofatumumab group (44% vs. 12%), the incidence of major hemorrhage was low and similar in the two groups (1% and 2%, respectively).^30^ In the phase III trial of ibrutinib-BR vs. placebo-BR, the rates of bleeding were 31% and 15%, respectively, and of major bleeding 2.1% and 1.7%, respectively. About 40% of patients in each arm were receiving anticoagulant or antiplatelet therapy.^36^ In the recently published study of first-line ibrutinib in elderly patients with newly diagnosed CLL, severe treatment-emergent bleeding was recorded in 4% of patients and led to treatment discontinuation in half of them.^37^

#### Cardiovascular toxicity: Atrial fibrillation and hypertension

An excess risk of atrial fibrillation (AF) was suggested by the results of early studies of ibrutinib in patients with CLL and mantle cell lymphoma, and was later confirmed in phase III randomized trials. The etiology of this AE remains largely unknown. A recent report suggested that AF might be related to an on-target effect of ibrutinib on BTK and related Tec kinase.^50^ One of the pathways regulated by BTK and Tec Kinase is the phosphoinositide 3-kinase (PI3K)-Akt pathway, which mediates cardiac protection under stress conditions.^51^ Reduced cardiac PI3K-Akt activity was found in specimens from patients with AF.^52^ In rat ventricular myocytes, therapeutic doses of ibrutinib caused reduced PI3K protein expression and Akt activation.^50^ Evidence of direct inhibition of PI3K activity by ibrutinib, direct involvement of BTK in the PI3K-Akt pathway, or direct cellular effects of ibrutinib on myocytes, however, is lacking.^53^

In the phase III trial, the incidence of AF was 3% vs. 0% in patients treated with ibrutinib vs. ofatumumab, respectively.^30^ In this study AF events were typically manageable, and led to treatment discontinuation in only one patient.^29^ The proportion of patients with a prior history of AF in the study was higher in the ibrutinib arm (5.6%) vs. ofatumumab (2.6%), suggesting that history of AF might be a risk factor for treatment-emergent AF during ibrutinib therapy.^30^ In line with these observations, the incidence of any-grade and severe AF in a phase III trial were 7.3% and 2.8%, respectively, in patients treated with ibrutinib plus BR, and 2.8% and 0.7%, respectively, in those receiving BR alone. One third of patients who developed AF temporarily interrupted treatment, and four permanently discontinued ibrutinib. Among patients with a prior history of arrhythmia, the rate of AF was 28% vs. 10% in the ibrutinib plus BR vs. BR group, respectively, thus confirming a higher incidence in patients with prior history of AF.^36^ In an analysis of their phase II study at a median follow-up of 28 months, Farooqui and colleagues found that AF occurred in 16% of patients during the study period, and was of grade ≥3 in 20% of them. In all but one patient, a trigger for AF could not be identified. The annualized incidence was 0.052 events per person-year of treatment without prior AF, which is higher than expected (0.0124) in the general population.^54^,^55^

Although additional data are needed, these results taken together suggest an increased risk of AF in patients with CLL treated with ibrutinib. One possible trigger for AF is hypertension. In the long-term follow-up analysis of the phase I study, treatment-emergent elevated blood pressure has been observed in 20% of patients with ibrutinib, particularly after 2 years of therapy.^29^,^37^

#### Neutropenia and other cytopenias

In the pivotal phase I study, around 20% of patients experienced grade 3–4 neutropenia, which was associated with fever in a quarter of them. Patients were generally managed with granulocyte-colony stimulating factor (G-CSF). Grade 3–4 anemia was observed in 5% of patients and was managed with erythropoietin stimulating agents. Cytopenias tended to occur early during the course of therapy and in no case led to treatment discontinuation.^27^ Similar figures and outcomes were observed in the phase III trial^30^ and other trials of ibrutinib monotherapy. One possible exception is represented by the study in patients with CLL and del(17p), wherein grade 3–4 neutropenia, anemia, and thrombocytopenia were observed in 24%, 14%, and 10% of patients, respectively.^31^

Clinically significant cytopenias tend to be short-lasting. In fact, some patients who presented with cytopenias before receiving ibrutinib improved their blood counts during treatment. For instance, in the study by Burger and colleagues, sustained improvement in cytopenias was noted in 15 (63%) patients with baseline thrombocytopenia, and 15 (88%) with anemia.^33^ Similar findings were reported in the phase III study of ibrutinib vs. chlorambucil in untreated elderly CLL patients.^37^ The lack of significant myelosuppression (and the presence of marrow restoration in some cases) is extremely important in a patient population that may have already experienced cytopenia related to marrow involvement and/or chemotherapy-induced marrow suppression.

In studies of ibrutinib in combination with other agents, the rates of grade 3–4 neutropenia tended to be higher (up to 53% when used in combination with BR). Management consisted of the use of G-CSF, and only 1% of patients discontinued therapy because of it.^33^–^36^

Of interest, a recent pooled analysis that included 301 CLL patients treated with ibrutinib in clinical trials showed that the incidence of treatment-emergent autoimmune cytopenias was low (13/1000 patient-years) and 86% of patients who were receiving treatment for autoimmune cytopenia at the time of ibrutinib were able to discontinue such treatment. Importantly, patients receiving immune therapies for, or experiencing uncontrolled autoimmune cytopenias were not included in those studies.^56^

#### Infections

The most common infectious complications in patients with CLL involve the respiratory tract. The frequency and pattern of infectious complications in patients treated with ibrutinib appears to reflect what is typically observed in this patient population, rather than a drug-specific AE profile. The most commonly reported infection across ibrutinib studies was upper respiratory tract infection (URI),^27^,^28^,^30^,^31^,^33^–^35^,^37^ mostly self-resolving without the need for interrupting treatment. Again, serious infections occurred early in the course of therapy and the rate declined after the first 6 months. Pneumonia was the most common serious infectious AE. As one would expect, the frequency of infections tended to be higher in relapsed/refractory patients (51%) than in treatment-naïve patients (13%).^29^ Supportive care included the use of antibiotics, G-CSF, intravenous immunoglobulin supplementation, and antiviral prophylaxis. Herpes zoster reactivation was only occasionally observed.

In the phase III trial, infections of any grade were more frequent in the ibrutinib arm (70% vs. 54%), but the rate of grade 3–4 infections was not significantly different between the two study groups (24% vs. 22%).^30^ The studies of ibrutinib in combination with other agents confirmed the same pattern of infectious complications, URI and pneumonia being the most common ones.^31^,^33^,^35^ In the phase III trial of BR with or without ibrutinib, exposure-adjusted serious infection rates were similar in the two arms (2.4/100 patient–months).^36^

## Idelalisib: Safety Considerations

The most common AE reported during treatment with idelalisib were pyrexia, fatigue, nausea, chills, diarrhea, and skin rash. Serious AE were recorded in 36 (67%) and 44 (40%) patients in the phase I and phase III study, respectively. In the phase I trial, seven patients (13%) had a dose reduction at some time during the primary study due to severe, mostly drug-related toxicity. Of the 29 patients (54%) who discontinued therapy, 5 (9%) did so due to AE. Of the 23 patients who continued idelalisib in the extension study, 8 remained on therapy with a median dosing duration of 29 months, and 2 of the 15 patients who discontinued therapy did so because of AE. Eleven patients died during the study; 8 of them during the primary study, and all but one due to AE.^38^ In the phase III trial 9 patients (8%) discontinued idelalisib, and 6 of them did so due to gastrointestinal or cutaneous complications.^39^ In the phase I trial, a maximum tolerated dose was not identified (maximum administered dose was 350 mg twice daily).^38^ Most patients in both the phase I and phase III trials had at least one AE, generally of grade 1–2. As described below in more detail, fatal and/or severe diarrhea or colitis, hepatotoxicity, pneumonitis, and intestinal perforation have been associated with treatment with idelalisib. Prescribing information therefore contains a black-box warning for such events. Additional warnings are also included regarding cutaneous reactions, anaphylaxis, neutropenia, and embryo-fetal toxicity.^20^ An overview of common and relevant AE in patients with CLL treated with idelalisib alone or in combination with rituximab is offered in Table 2, and discussed below.

### AE of Special Interest

#### Diarrhea and colitis

Diarrhea, with or without colitis, may represent a class effect of PI3K inhibitors. Mice with inactivating mutations of P110δ PI3K developed an inflammatory bowel disease, mostly limited to the large intestine. Histologically, lesions were characterized by mucosal hyperplasia, crypt abscesses, and B- and T-leukocyte infiltrates.^57^ Idelalisib-related diarrhea can present with two different patterns. Early diarrhea occurs within the first 8 weeks of therapy, is typically mild, and responds to common antimotility agents. In contrast, late diarrhea can be of sudden onset and severe, and it associates with colitis. It is typically watery, without cramps, blood, or mucus. Histologically, the picture is consistent with lymphocytic colitis. This type of diarrhea typically does not respond to antidiarrheal agents or antibiotics, but tends to improve in most cases following treatment interruption.

The median time to onset is about two months for mild diarrhea and seven months for severe diarrhea.^58^ In the phase I study, diarrhea was reported in 30% of subjects and was of grade 3–4 in 6% of cases. Clinical evidence of colitis was present in 7% of these patients.^38^ In the randomized trial, diarrhea of any grade occurred in 21% of patients receiving idelalisib, and it was severe in 5% of cases.^39^ Interestingly, recent data on the use of idelalisib in treatment-naïve patients with CLL indicate further increased incidence of diarrhea/colitis. In a phase II study of 64 older patients treated with combined rituximab and idelalisib, any-grade and severe diarrhea were observed in 64% and 42% of patients, respectively, and severe colitis was observed in 25% of cases.^59^ In a separate study of 21 patients treated with combined idelalisib and ofatumumab, 76% of them experienced a grade ≥3 toxicity and 14% had enterocolitis. A higher incidence of grade ≥3 toxicities was found in younger patients with higher absolute lymphocyte counts.^60^ Five out of the 6 subjects studied with single-cell mass spectrometry were found to have decreased T regulatory cells (Tregs) after idelalisib therapy, and Tregs from subjects who experienced severe toxicity expressed both lower levels of functional markers and higher levels of apoptotic markers.^60^ Taken together, these data suggest, perhaps unsurprisingly, that treatment-naïve patients, whose immune system is more preserved, may be at higher risk for immune-related toxicities after idelalisib therapy.

According to most authors, in case of mild-moderate diarrhea (4–6 bowel movements daily), idelalisib treatment may be maintained with very close patient monitoring. Antidiarrheal agents should be used and the American Dietetic Association colitis diet implemented. In the case of severe diarrhea (≥7 bowel movements per day) or diarrhea qualifying as serious AE, idelalisib should be immediately withheld, and infectious etiology should be ruled out. Intravenous hydration and intraluminal or systemic corticosteroids should be considered to expedite resolution. The patient should be monitored until resolution, at which point idelalisib treatment may be resumed at a reduced dose of 100 mg twice daily. Steroid therapy may be maintained after treatment resumption to control diarrhea and allow for continued idelalisib therapy. If, however, the patient experiences grade 4 diarrhea, idelalisib should be permanently discontinued.^58^

#### Hepatotoxicity

Elevation in alanine transaminase (ALT) and aspartate transaminase (AST) levels can occur during treatment with idelalisib. In fact, hepatotoxicity was one of the most common reasons for discontinuation of the drug in clinical trials. It typically manifests 4–12 weeks after treatment initiation in asymptomatic patients. This toxicity is reversible in the majority of cases after dose interruption, and about 75% of patients do not experience recurrence upon resumption at a lower dose. Grade 3–4 elevation of ALT or AST (>5 times the ULN), however, has been reported in 14% of patients, and led to death in one case.^58^

In the phase I trial, 28% of patients experienced liver enzyme elevation of any grade, and only one had a grade 3 hepatotoxicity.^38^ In the phase III study, transaminase level elevations were more common in the idelalisib plus rituximab arm. Grade 3 or higher elevations occurred in 5–8% of patients and an increase of any grade occurred in 25–35% in the idelalisib group. The study drug was withheld and successfully reinitiated in two thirds of these patients.^39^

The idelalisib prescribing information recommends that it not be taken concomitantly with drugs of hepatotoxic potential, and advises close monitoring of ALT and AST in the first six months of treatment. For AST/ALT elevations 3–5 times the upper limit of normal (ULN), idelalisib can be maintained with closer patient monitoring. For levels 5–20 times the ULN, the drug should be interrupted and the patient monitored closely until levels return below the ULN. For elevations >20 times the ULN, however, idelalisib should be permanently discontinued.^58^ Again, in frontline studies of idelalisib, elevated ALT and/or AST levels were observed in a greater proportion of patients. Grade ≥3 elevations occurred in 67% and 57% of patients in the trials from O’Brien^59^ and Lampson,^60^ respectively. In the latter study, liver biopsies showed increased activated cytotoxic T cells within the liver parenchyma.

#### Pneumonia and pneumonitis

Infectious or non-infectious lung injury has been reported in patients treated with idelalisib.^20^ Among 24 cases observed across clinical trials, 19 were serious and three resulted in death.^58^ In the phase I study, pneumonia was reported in 20% of the patients. All the events were of grade ≥3 and included bacterial and fungal (including *Pneumocystis jirovecii*) etiologies. Three of these patients had organizing pneumonia (2) or interstitial pneumonitis (1). Each was treated with steroids and two were able to continue therapy after resolution.^38^ In the phase III combination study, 4% of patients in the idelalisib plus rituximab arm experienced pneumonia; no grade 3–4 cases, however, were reported.^39^ These data contrast with results from the ibrutinib experience, where non-infectious pneumonitis has only been reported anecdotally.^61^ In general, each patient presenting with signs or symptoms suggestive of lung injury should be immediately and thoroughly evaluated. If an infectious source cannot be identified, a course of systemic steroids in addition to antibiotics should be administered. In every case, prompt recognition and management are paramount to minimize severity and reduce the risk of mortality.

#### Neutropenia and other cytopenias

Treatment-emergent severe neutropenia has been reported in 34–43% of patients with CLL treated with idelalisib in clinical trials. G-CSF was used in about a quarter of the patients in the idelalisib plus rituximab cohort, in the phase III trial.^39^

In general, grade 3–4 anemia and thrombocytopenia were largely transient in patients with pre-existing hematologic abnormalities. Furthermore, almost all patients with severe cytopenias at baseline improved or normalized their counts during the course of treatment.^38^,^39^ Amelioration of cytopenias was also recently confirmed in a pooled analysis of two phase III randomized trials of rituximab with or without idelalisib in patients with CLL and other types of indolent non-Hodgkin lymphoma, respectively.^62^

#### Infections

The infectious toxicity profile of idelalisib, alone or in combination with other agents, was in line with the expected pattern of events seen in a population of patients with CLL who had received several lines of therapy. As mentioned above, one of the most common severe infections in patients treated with idelalisib is pneumonia (7–20%), followed by febrile neutropenia (5–11%), bacteremia/sepsis (6–7%), and cellulitis (1–6%). Among the 164 patients treated in the phase I and phase III studies, five (3%) had *Pneumocystis jirovecii* pneumonia (one patient was believed to have contracted it prior to treatment initiation) and one developed cytomegalovirus reactivation. Idelalisib treatment was not associated with significant decrease in serum immunoglobulin levels or changes in T-cell subpopulation distribution.^38^,^39^

## Conclusions

The advent of BCR signaling inhibitors has revolutionized the treatment of CLL, as well as other chronic lymphoproliferative neoplasms, in many respects. These agents have shown efficacy in previously treated and treatment-naïve patients. They are administered orally, which is often seen as favorable from a patient perspective, and, even more importantly, they are characterized by a favorable toxicity profile. Early results of a newer, more selective BTK inhibitor, acalabrutinib, suggest that this agent may be devoid of many of the potentially serious toxicities associated with ibrutinib, including hemorrhage and AF.^63^ These features are especially valuable in a population that includes a large proportion of older and, at times, frail patients. The mechanisms of some of the most concerning side effects are being studied, and, as more knowledge is gained, new precautions may be devised to help prevent or decrease the rate of serious complications. As more patients are treated on clinical trials and in clinical practice, the safety profile of BCR signaling inhibitors will be better defined and management of AE is likely to improve. Clinicians, however, should consider that, since these agents are administered continuously and indefinitely, the impact of AE (including mild ones) on patients’ quality of life and compliance may be greater than with short-term treatments.

Furthermore, long-term data on their safety are limited at the present time. Future updates of ongoing and completed studies and post-marketing analyses will certainly help better evaluate the safety of these drugs.

## Figures and Tables

**Table 1:** Selected adverse events in patients with chronic lymphocytic leukemia treated with ibrutinib alone or in combination with other agents in clinical trials.

	Ibrutinib monotherapy^27^–^31^,^37^,^55^		Ibrutinib + anti-CD20 mAb^33^,^34^		Ibrutinib + bendamustine-rituximab^36^,^38^	
	% any grade	% grade ≥3	% any grade	% grade ≥3	% any grade	% grade ≥3
**AE of special interest:**						
Bleeding	44–69	1–8	36–48	3–10	3–31	3–4
Hypertension	14–23	4–20				
Atrial fibrillation	6–16	2–6	6	0–6	0–7	0–1
Cytopenias:						
Neutropenia	16–30	10–24	24	6–24	40–59	40–54
Anemia	16–23	0–14	8–16	0	22	0–3
Thrombocytopenia	16–21	5–10	0	0	17–31	7–15
Infections (selected):						
Pneumonia	10–20	4–20	20–41	5–17	13	8
URI	16–26	0–2	27–37	0–3	16–37	2–3
Sinusitis	2–23	1–5	16	0	27	0
Cellulitis	0–17	0–4			7	7
UTI	0–10	0–4	8	0	3	3
Sepsis	0–5	0–5	3	3		
Fever (non-neutropenic)	4–24	0–5			23–24	0–3
Neutropenic fever	0–2	0–2			7–12	7–12
**Other AE (selected):**						
Diarrhea	42–61	0–6	26–70	0–7	35–70	2–3
Arthralgia	16–59	0–1	17–28	0	11–27	1–3
Muscle pain	13–37	1–2	40	0	11–40	0
Cough	19–31	0			19	0
Fatigue	5–30	0–5	18–21	0–3	21–47	3–10
Nausea/vomiting	6–26	0–2	24–28	0	37–67	1–3
Rash	3–47	0–3			17–18	1–13
Edema	8–29	0–1	18	0	12–33	1–3
Constipation	15–16	0–1	11	0	18–30	0
Eye disorders	10–17	0			23–30	0–1
Back pain	11–16	0–3			11–17	0–1
Stomatitis	11–16	0–1	38	3		
Headache	4–22	1–3	8	0	15–30	0–2
Peripheral neuropathy	4–13	0	9–44	3		

Abbreviations: mAb, monoclonal antibody; AE, adverse event; URI, upper respiratory infection, UTI, urinary tract infection.

**Table 2:** Selected adverse events in patients with chronic lymphocytic leukemia treated with idelalisib alone or in combination with rituximab in clinical trials.^38^,^39^,^59^

	% any grade	% grade ≥3
**AE of special interest:**		
Pneumonitis	3–4	3–4
Diarrhea/colitis	19–64	4–42
ALT or AST elevation	35	5
AP elevation	33	0
GGT elevation	20	2
Bilirubin elevation	26	0
Cytopenias:		
Neutropenia	55–57	34–43
Anemia	25–37	5–11
Thrombocytopenia	17–30	10–17
Infections (selected):		
Pneumonia	9–28	9–20
URI	22	
Cellulitis	1–6	1–6
Bacteremia	6	6
Sepsis	7	7
Sinusitis	11	2
UTI	16	6
Fever (non neutropenic)	28–42	3–4
Neutropenic fever	5–11	5–11
**Other AE (selected):**		
Hypertriglyceridemia	56	4
Hyperglycemia	46	6
Hypocalcemia	44	4
Hypernatremia	30	4
Fatigue	24–31	2–3
Hyoglycemia	26	4
Nausea/vomiting	24–38	0–3
Back pain	16–22	2
Chills	17–36	2
Cough	15–33	0–4
Rash	10–58	2–13
Headache	13–23	0
Constipation	12–17	0
Arthralgia	11–17	0–2
Edema	11	0

Abbreviations: AE, adverse event; ALT, alanine aminotransferase; AST, aspartate aminotransferase; AP, alkaline phosphatase; GGT, gamma glutamyltransferase; URI, upper respiratory infection; UTI, urinary tract infection.
